# Hand washing practice among public primary school children and associated factors in Harar town, eastern Ethiopia: An institution-based cross-sectional study

**DOI:** 10.3389/fpubh.2022.975507

**Published:** 2022-11-03

**Authors:** Ashenafi Berhanu, Dechasa Adare Mengistu, Liku Muche Temesgen, Salie Mulat, Gebisa Dirirsa, Fekade Ketema Alemu, Adane Ermias Mangasha, Tesfaye Gobena, Abraham Geremew

**Affiliations:** ^1^Department Environmental Health Sciences, College of Health and Medical Sciences, Haramaya University, Harar, Ethiopia; ^2^School of Public and Environmental Health, Hawassa University, Hawassa, Ethiopia

**Keywords:** infectious disease, hand washing, practice, school-aged children, Ethiopia

## Abstract

**Background:**

Hand washing with soap and water reduces the risk of diarrheal episode by 28–48% and acute respiratory infection by 20–50%. However, there is limited evidence on hand washing practices among students in Eastern Ethiopia, particularly in Harari town. Therefore, this study aimed to determine hand washing practice among primary school students and associated factors in Harar town, Eastern Ethiopia.

**Methods:**

An institution-based cross-sectional study was applied among 670 students in Harar town from June 1 to 30, 2021. A multi-stage sampling was employed; 6 out of 20 schools were selected through simple random sampling, while eligible children from each school was selected by probability proportional to size sampling method. Data were collected using a pre-tested questionnaire with a face-to-face interview technique and via observation. The data were analyzed using SPSS software version 23. Binary and mult-variable analysis were used to determine the association between factors and outcome variable. Finally, a *p*-value of < 0.05 was considered to declare a statistically significant association.

**Results:**

A total of 670 participants were included in the study, of which 248 (37.0%) had washed their hands [95% CI: 33.3–40.06]. Being in grade 8 Adjusted Odd Ratio[AOR = 4.9; 95% Confidence Interval (CI): 2.28–10.52], living in an urban area [AOR = 3.49; 95% CI: 1.29–9.40], having role models (parents [AOR = 4.41; 95% CI: 1.79–10.86], teachers [AOR = 3.69; 95% CI: 1.39–8.81], and health professionals [AOR = 3.17, 95% CI: 1.17–8.63]), availability of hand washing facility [AOR = 3.62; 95% CI: 1.57–8.34], access to soap and water [AOR = 2.89; 95% CI: 1.39–5.98] and being membership of water sanitation and hygiene (WASH) club [AOR = 2.39; 95% CI: 1.41–4.03] were found to be significantly associated with hand washing practice.

**Conclusions:**

The current study found that nearly a third of students practiced proper hand washing. Hand washing practice was influenced by students' grade level, residence, referents (role models for hand washing), presence of a hand washing facility, access to water and soap, and membership of WASH club. Therefore, the finding revealed that there is a need to improve hand-washing practices in schools by concerned agencies.

## Introduction

Handwashing with soap is a specific action of hand hygiene that involves the use of soap and water to physically remove dirt, organic material, and microorganisms from hands (1). It is recognized and accepted as a low-cost and effective technique for preventing communicable diseases (2). Research suggests that handwashing with soap can reduce diarrheal episodes by 28 to 47% and can reduce acute respiratory infections, such as pneumonia, by 20 to 50% (3–8). Handwashing can also limit disease outbreaks, such as cholera and Ebola, and reduce healthcare-associated infections by more than 50% (9). Moreover, Global Handwashing Day was dedicated to increasing awareness and understanding of the importance of hand washing with soap at a critical time as an effective and affordable way to prevent diseases (2).

School-aged children in developing countries do not usually engage in hand washing practice at critical times, such as after using the toilet, before eating, and before cooking the food (10, 11). In addition, due to the close proximity of children in schools and child care settings, there is a high risk of the spread of infectious diseases. However, less than 5% of the population in developing countries still practices hand washing (12). Therefore, to protect the health and well-being of children, it is important to prevent infectious diseases such as diarrhea, pneumonia, and other communicable diseases (13, 14). However, about 400 million children are infected with worms as a result of poor hand washing practices (15).

Furthermore, proper hand washing contributes to improve the learning and teaching processes by reducing absenteeism due to illness, for instance, about 45, 40, 35, 27, and 20% of school absenteeism is reported in China, Egypt, Kenya, Philippines, and Colombia, respectively (16). About 443 million school days are lost each year due to water-related illnesses, making it a leading factor in school absenteeism in the developing world (2). Although national initiatives through the school WASH program have been implemented to improve the hygiene status of schools and enhance the handwashing practices of students (17), more robust and better-designed interventions are needed to increase student hand washing practices.

Moreover, adequate water supply and hygiene conditions are low in many schools in Ethiopia. Besides these problems, there is limited evidence regarding handwashing among schoolchildren. Similarly, there is no regular supervision and adequate hand-washing facilities in most schools, which can increase the risk of disease transmission. Besides these problems, there is limited evidence regarding handwashing practices in Ethiopia, including Harar town. Therefore, this study aimed to determine hand washing practice among primary school students and associated factors in Harar town, eastern Ethiopia.

## Materials and methods

### Study setting, design and period

An institution-based quantitative cross-sectional study was conducted in Harari regional state, Harar town, eastern Ethiopia from June 1 to June 30, 2021. The town is located in 526 km away, toward the east of Addis Ababa, the capital city of Ethiopia. Administratively, it is divided into 19 kebeles (the lowest administrative division in the country). The average temperature of the town is 23°C, with an estimated area of 334 square kilometers. According to the 2007 census of Ethiopia, the Harari region had a total estimated population of 183,415 people, of which 92,316 were men and 91,099 were women (18). In this town, there are 20 public primary schools. These public primary schools provide education for about 7,661 students in Harar town and nearby rural areas in the 2020/21 academic year.

### Source and study population

All students registered in primary schools in Harar town in the 2021 academic year were a source population. All students attending grades 5–8 in selected schools in Harar town during the 2021 academic year were study population.

### Inclusion and exclusion criteria

All students from grades 5–8 from selected public primary schools who were registered and attending class during data collection were included in the study. Students with speech and hearing impairments, learning disabilities, and those taking night classes were excluded from the study.

### Sample size determination

The required sample size was calculated by using a single population proportion formula as follows by considering a previous prevalence of *P* = 28.1%, the proportion of primary school students who practice proper hand washing practices in Ethiopia (19).


$$\begin{array}{l} \text{n}=\frac{{\left(\text{z}\alpha /2\right)}^{2}*\text{P}\left(1-\text{P}\right)}{{\text{d}}^{2}} \end{array}$$

Where *N* is the required minimum sample size, Z is the value at 95% confidence intervals (1.96), and the margin of error is 5%. A design effect of two was considered. By considering the non-response rate (10%), a total sample size of 682 participants were included in this study.

### Sampling procedure and technique

**Figure 1:**
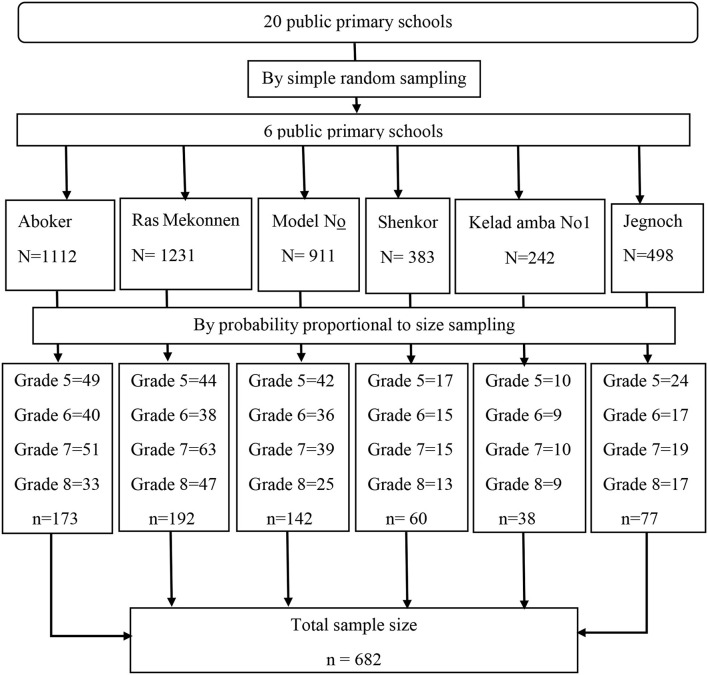
A schematic presentation of sampling procedure for the study to assess magnitude of hand washing practice among primary school children and associated factors in Harar town, eastern Ethiopia, 2021.

A multi-stage sampling procedure was employed to select study participants. In the first stage, 6 out of 20 primary schools were selected through random sampling, while eligible children from each school was selected by probability proportional to size sampling method by using the following formula: *n*_i =_ *n* ^*^ *N*_i/_*N*; *n*_i_ – required sample size from each grade, *n*–total calculated sample size, *N*_i_ – total number of students in each grade and *N*–total number of students of in 6 primary schools. Among 682 selected; 173, 19*2*, 14*2*, 60, 38 and 77 students were selected from Aboker, Ras Mekonnen, Model *N*°1, Shenkor, Kelad amba *N*°1 and Jegnoch school respectively). A list of students served as a sampling frame, and study participants were selected using a table of random numbers (Figure 1).

### Data collection methods

A pre-tested questionnaire, a face-to-face interview technique, and an observational checklist were used to collect data. Prior to data collection, training was given for data collators on the objective, tools, ethics, and techniques of data collection for the study. The questionnaire was adapted from different literature (19–21) and the WHO survey of school WASH programs. The English version of the questionnaire was translated into Amharic and Afan Oromo (the local language of the study participants). The questionnaire consisted of five parts enquiring about students' hand washing practices and associated factors. The first part includes socio-demographic information about the students and parents. This part of the study consists of age, sex, grade, residence, educational level of parents, and occupation of parents. The second part is about knowledge and attitude. The third part is about social factors (such as important referents and sources of information). The fourth part is about personal factors and the fifth part is about facility-level factors (including availability and accessibility of soap, water, and hand-washing facilities).

### Study variables

The dependent variable for this study was hand washing practice, while socio-demographic factors (such as age, gender, grade, residence, parents' educational status, and parents' occupation), knowledge and attitude, social factors (such as important referents (role models) and sources of information), personal factors, facility-level factors (such as availability and accessibility of soap and water), and facility-level factors (such as availability and accessibility of soap and water) were independent variables.

### Operational definitions

**Hand washing at a critical time:** includes washing hands before preparing food, before having a meal, after eating, and after visiting the rest room (22).

To assess the level of hand washing practices, respondents were asked 7 questions, and those who scored mean or above on practice questions were considered to have proper practices, and those who scored less than mean were considered to have improper practices (19, 23, 24).

Hand washing knowledge was assessed based on 8 questions. Those who scored mean or above were classified as having good knowledge, and those who scored less than mean were classified as having poor knowledge (20, 21). To assess attitudes toward hand washing, respondents were asked 4 attitude questions with 5-scale Likert items. Those study participants who scored mean or above on the attitude questions were considered to have a positive attitude toward hand washing practice, and those who scored less than the mean were classified as having a negative attitude (23, 25).

**Hygiene facility**: School hygiene status was assessed by combining three hygiene facilities in schools (access to water, soap, and hand washing station). Schools that had all three facilities were categorized as offering basic hygiene services, while those with only two facilities (hand washing station with water but no soap available) were categorized as offering limited services, and schools without any hand washing stations or water at the time of data collection were categorized as offering no hygiene services (26).

### Data quality control

Before data collection, the tool was pre-tested on 5% of the sample in Sabian primary school in Dire Dawa to check its validity. Based on the findings from the pretest, the data collection tool was revised, edited, and the necessary corrections were made before actual data collection. Either the Amharic and Afan Oromo version of the questionnaire was interviewed based on the preference of the respondents. Two-day training was given for data collectors and supervisors on the objectives, confidentiality of Information, and data collection techniques of the study. Follow-up and supervision of data collectors was done during data collection. The filled questionnaire was checked for completeness and consistency of response by two trained supervisors on a daily basis.

### Data processing and analysis

The data was coded, edited, cleaned, and entered into Epi-Data 4.6.1, then exported to SPSS 23 for data analysis. A descriptive statistical analysis was used to describe the characteristics of study participants using frequency and tables. Crude and adjusted odds ratios were computed using logistic regression to identify the association between outcome variable and factors. The model's goodness of fit was checked by using the Hosmer-Lemeshow statistic test. Multicollinearity among independent variables was checked using the variance inflation factor and tolerance test. The odds ratio and 95% confidence interval were used to determine the direction and strength of the statistical association. Finally, a *p*-value < 0.05 was considered to declare statistically significant association.

## Results

### Socio-demographic characteristics of respondents

Out of 682 study participants, 670 participated in this study with a response rate of 98.3%. Of the total participants, 185 (27.6%), 149 (22.2), 192 (28.7%) and 144 **(**21.5%) were from grade 5, grade 6, grade 7 and grade 8 respectively. Furthermore, more than 381 (56.9%) study participants were females and majority 555 (82.7%) were urban residents. The mean age of the study participants was 14.05 years (SD = 1.70) (Table 1).

**Table 1:** Socio-demographic characteristics of primary school children in Harar town, eastern Ethiopia 2021(*n* = 670).

**Variables**	**Categories**	**Frequency**	**Percent**
Grade	Grade 5	185	27.6
	Grade 6	149	22.2
	Grade 7	192	28.7
	Grade 8	144	21.5
Age	<14	264	39.4
	≥14	406	60.6
Sex	Male	289	43.1
	Female	381	56.9
Residence	Rural	117	17.3
	Urban	555	82.7
Mothers education	No formal education	119	17.8
	Read and write	133	19.9
	Grade 1–8	229	34.2
	Grade 9–12	85	12.7
	>Grade 12	104	15.5
Mothers occupation	House wife	288	43.0
	Non-house wife^*^	382	57.0
Fathers education	No formal education	58	8.7
	Read and write	123	18.4
	Grade 1–8	181	27.0
	Grade 9–12	119	17.8
	>Grade 12	189	28.2
Fathers occupation	Farmer	105	15.7
	Non farmer^*^	565	84.3

* Government Employed, Private employed, Merchant, Laborer.

### Hand washing practice among students

Of total study participants, nearly a third 248 (37.0%, 95% CI (33.3–40.06), had good hand washing practice. The majority (85.1%) of students had washed their hands in the morning of interview day. Among total respondents, 349 (52.1%) and 193 (28.3%) had used only plain water and water with soap to wash their hands, respectively. About 351 (52.4%) and 151 (22.5%) spent less than 20 seconds for washing their hands at a time and didn't know how long to wash their hands at a time, respectively (Table 2).

**Table 2:** Hand washing practice of primary school children in Harar town, eastern Ethiopia 2021(*n* = 670).

**Variables**	**Categories**	**Frequency**	**Percent**
Have you washed your hands in the last 12 hours	Yes	570	85.1
	No	100	14.9
Usual hand washing time	Before meal	344	51.3
	After meal	154	23.0
	After work	52	7.8
	After play	31	4.6
	After toilet	89	13.3
Item used for hand washing (*n* = 570)	Water only	349	52.1
	Water and soap	193	28.8
	Water with ash	20	2.7
More practiced to wash hand in the family	Water only	445	66.4
	Soap and water	215	32.1
	Water and ash	10	1.5
Duration of hand washing at a time	<20 S	351	52.4
	20 S−1 min	162	25.1
	Don't know	151	22.5
How often do wash hand with soap before meal	Always	168	25.1
	Very often	78	11.6
	Often	350	52.2
	Sometimes	69	10.3
How often do wash hand with soap after toilet	Always	84	12.5
	Very often	66	9.9
	Often	377	56.3
	Sometimes	125	18.7
Proper hand washing practice	Yes	248	37.0
	No	422	63.0

### Students' knowledge and attitude about hand washing practice

The current study revealed that more than half, 413 (61.6%) of students had good knowledge about hand washing practice. Furthermore, about two-thirds 446 (66.6%) of students had a positive attitude (Table 3).

**Table 3:** Students' knowledge and attitude about hand washing practice in Harar town, eastern Ethiopia 2021(*n* = 670).

**Variables**	**Categories**	**Frequency**	**Percent**
Do human feces contain germs	Yes	590	88.1
	No	80	11.9
All clean objects are free from germ	Yes	411	61.3
	No	259	38.7
Human urine contain germ	Yes	557	83.1
	No	113	16.9
Germs can be acquired when desks, doors books and animals are touched	Yes	502	74.9
	No	168	25.1
Poor hand washing cause disease	Yes	610	91.0
	No	60	9.0
Water only enough for hand washing	Yes	147	21.9
	No	523	78.1
Hand washing with soap needed after sneezing	Yes	537	80.1
	No	133	19.9
Failure to wash hand transmit infectious disease	Yes	615	91.8
	No	55	8.2
Overall knowledge	Good	413	61.6
	Poor	257	38.40
If you wash your hands really well with water you don't need to use soap	Yes	508	75.80
	No	162	24.20
You only need to wash your hands with soap if they look dirty or smell bad	Yes	450	67.20
	No	220	32.80
Young infant's feces do not contain germs	Yes	500	74.60
	No	170	25.40
Washing your hands with soap before feeding is important only if you use your hands to feed	Yes	481	71.80
	No	189	28.20
Overall attitude	Positive	446	66.60
	Negative	224	33.40

### Personal level factors about hand washing practice

**Figure 2:**
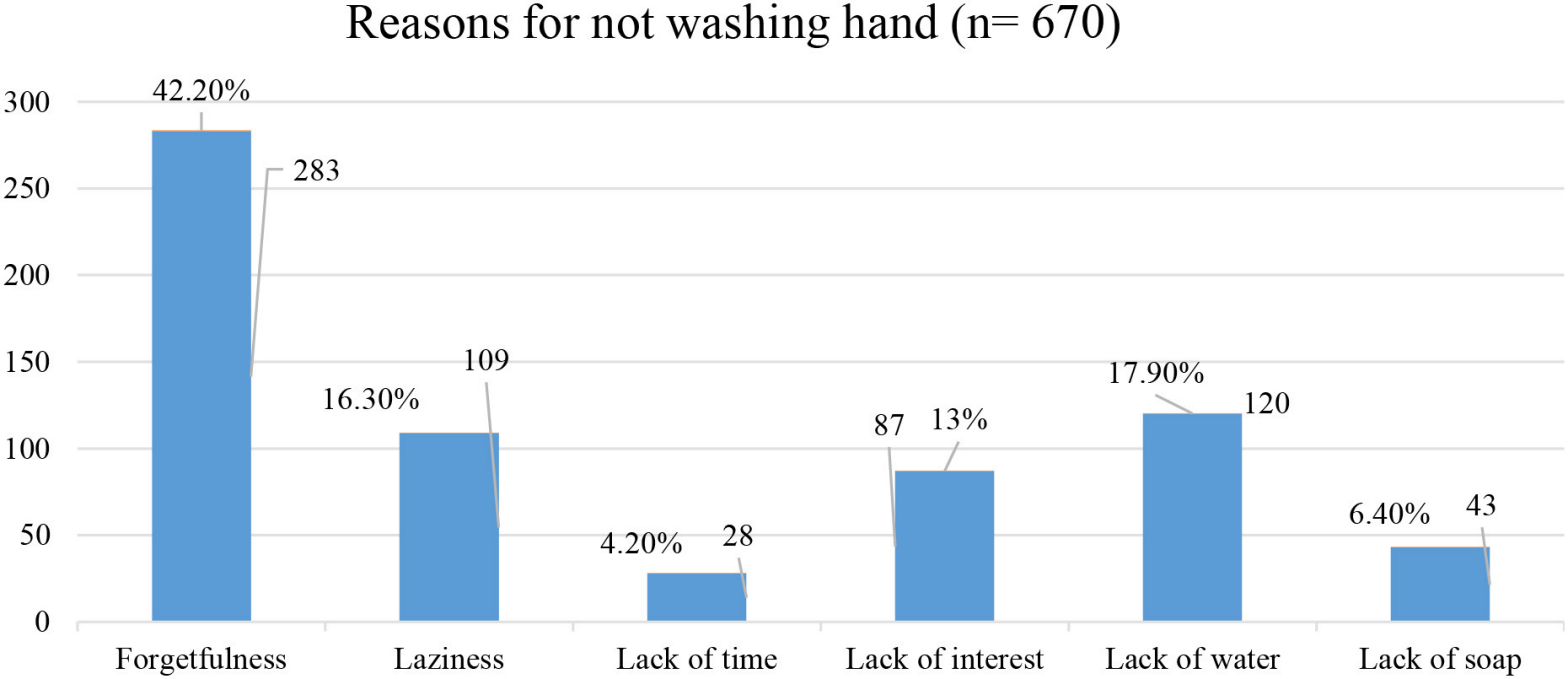
Reported reasons for not washing hands in Harar town, eastern Ethiopia 2021.

The majority 516 (77.0%) of the study participants had reported that the benefit of hand washing is for disease prevention, while 17 (2.5%), 54 (8.1%) 83 (12.4%) mentioned that as they wash their hands to remove bad smell, to remove dirties and to clean hands respectively. Moreover, forgetfulness is the main reported reason for not washing their hands (Figure 2).

### Availability of hand washing facilities

Based on the observational finding, five out of six schools had hand washing facilities in their compound within walking distance. About four schools had provided water, but none of the schools had soap for hand washing. Regarding school hygiene facilities, only five schools fulfilled criteria for limited hygiene facilities, but none of the schools fulfilled criteria for basic hygiene facilities (Table 4).

**Table 4:** Availability of hand washing facilities in primary schools in Harar town, eastern Ethiopia 2021.

**Variable**	**Categories**	**Frequency**	**Percent**
Is there hand washing facility in school?	Yes	5	83.30
	No	1	16.70
Location of hand washing facilities?	Next to latrines	2	33.33
	Within walking distance	4	66.66
What kind of facility?	Reservoir and faucet (piped)	5	100.00
	Bucket (hand poured)	0	
Is water available for hand washing?	Yes	4	66.6
	No	2	33.33
Does hand washing facility have soap near to it?	Yes	0	
	No	6	100.00
Does the school have Hygiene and sanitation club?	Yes	349	52.10
	No	321	47.90
Are you member of wash club?	Yes	172	25.70
	No	177	26.40
Does the school celebrated hand washing day?	Yes	303	45.20
	No	367	54.80
Does school have any reminder for students to wash their hands?	Yes	4	66.66
	No	2	33.33
Is there any hand promotion activities?	Yes	3	50.00
	No	3	50.00
School hygiene facility	Basic	0	
	Limited	5	83.30
	No Facility	1	

### Social level factors (information level factors) of hand washing practice

Regarding information about hand washing, nearly three-quarter (73.6%) of students reported that they had heard information from television, which is the main source of information mentioned by students. Furthermore, 292 (43.6%) study participants reported that parents were their role models to wash hands soap (Table 5).

**Table 5:** Social level factors (information level factors) of children in primary school in Harar town, eastern Ethiopia 2021(*n* = 670).

**Variable**	**Response**	**Frequency**	**Percent**
Sources of information	Television	493	73.6
	Radio	86	12.8
	Leaflets	17	2.5
	News papers	24	3.6
	Mini-media	25	3.7
	Others	25	3.7
Referents for hand washing	Parents	292	43.6
	Teachers	138	20.6
	Health professionals	128	19.1
	Friends	112	16.7

### Factors associated with hand washing practice

A bivariate logistic regression analysis identified a significant association between the proper hand washing practice and grade of the respondents, sex, residence, educational level of mother, having referents (role model person) as parents, teachers, and health professionals, presence of a hand washing facility in a student's home, availability of water and soap for hand washing at home, and availability of a WASH club in school; being a member of the WASH club, and knowledge of participants.

The multi-variable regression shows that the odds of hand washing practices among students in grade eight were 4.9 times higher compared to grade five (AOR = 4.9, 95% CI: 2.28–10.52). Place of residence is a significant predictor of proper hand washing practice, whereby the odds of hand washing practice among students of urban residents was 3.5 times higher compared to their counterparts [AOR = 3.49, 95% CI: 1.29–9.40]. Respondents whose referents were parents, teachers, and health professionals were more likely to practice proper hand washing compared to those whose referents were friends [AOR = 4.41, 95% CI: 1.79–10.86], [AOR = 3.69, 95% CI: 1.39–8.81] and [AOR = 3.17, 95% CI: 1.17–8.63], respectively.

The findings of our study revealed that the odds of hand washing practice were 3.62 times higher among students who had a hand washing facility in their home compared to their counterparts [AOR = 3.62, 95% CI: 1.57–8.34]. Regular access to soap and water at a student's home is also significantly associated with proper hand washing practice, whereby students who had access to soap and water at home were about 2.9 times more likely to practice proper hand washing [AOR 2.89, 95% CI: 1.39–5.98]. Students who were members of the WASH club had 2.4 times higher chances of proper hand washing practice compared to their counterparts [AOR = 2.39, 95% CI: 1.41–4.03] (Table 6).

**Table 6:** Factors associated with hand washing practice among primary school students in Harar town, eastern Ethiopia 2021(*n* = 670).

**Variables**	**Response**	**Hand washing Practice**		**COR (95% CI)**	**AOR (95% CI)**	***p-*value**
		**Proper**	**Improper**			
Grade	Grade 5	34	151	1	1	
	Grade 6	48	101	2.11 (1.27–3.50)	1.16 (0.53–1.79)	0.707
	Grade 7	78	114	3.03 (1.89–4.86)	1.32 (0.67–2.60)	0.413
	Grade 8	88	56	6.97(4.23–11.51)	4.90 (2.28–10.52)	0.001
Sex	Male	94	195	1	1	
	Female	154	227	1.41 (1.02–1.93)	1.06 (0.63–1.79)	0.816
Residence	Rural	14	103	1	1	
	Urban	234	319	5.39 (3.01–9.67)	3.49 (1.29–9.40)	0.013
Mothers education	No formal education	32	87	1	1	
	Read and write	40	93	1.16 (0.68–2.02)	1.07 (0.69–4.00)	0.878
	Grade 1-8	91	93	1.79 (1.10-2.90)	1.66 (0.42–2.71)	0.257
	Grade 9-12	35	50	1.90 (1.05–3.44)	2.15 (0.98–4.67)	0.062
	> Grade 12	50	54	2.51 (1.44–1.40)	2.28 (0.960–5.45)	0.057
Referents	Parents	127	165	3.35 (1.96–5.65)	4.41 (1.79–10.86)	0.001
	Teachers	54	84	2.78 (1.55–4.99)	3.69 (1.39–8.81)	0.009
	Health Professionals	46	82	2.43 (1.33–4.41)	3.17 (1.17–8.63)	0.023
	Friends	21	91	1	1	
Presence of Hand washing station	Yes	223	247	6.32 (4.00–9.97)	3.62 (1.57–8.34)	0.002
	No	25	175	1	1.00	
Availability of soap and water	Yes	192	277	1.79 (1.25–2.57)	2.89 (1.39–5.98)	0.004
	No	56	145	1	1	
Availability of WASH Club	Yes	147	202	1.58 (1.15–2.17)	1.52 (0.12–1.87)	0.743
	No	101	220	1	1	
Membership of WASH club	Yes	92	80	2.84 (1.82–4.42)	2.39 (1.41–4.03)	0.001
	No	51	126	1	1	
Knowledge	Good	168	245	1.51 (1.09–2.10)	1.73 (0.98–3.05)	0.057
	Poor	80	177	1	1	

COR: Crude Odds Ratio; AOR: Adjusted Odds Ratio; CI: Confidence Interval.

## Discussion

Hand washing is remarkably essential for controlling infections, and schools are considered the right place to initiate this practice, starting from in childhood. The result of this study revealed that nearly thirds (37.0% [95% CI: 33.3–40.06]) of students practiced proper hand washing in primary schools in Harar Town, eastern Ethiopia.

The finding of our study was a bit higher than other studies conducted in Wolaita zone, Ethiopia, which was (28.1%) (19), Arbaminch town, Ethiopia, which was (22.3%) (20) and Sebata town, Ethiopia, which was (32.8%) (21). The finding from our study was in line with a study conducted in Colombia, which found that (33.6%) (27). However, it was a slightly lower proportion compared to studies conducted in India, which was (44.19%) (28) and in Wuhan, China, which was 42.05% (29). The possible reasons for this variation in proportion might be the results of socio-demographic, economic, and behavioral differences; increased access to hand washing facilities both in schools and homes; and many promotion activities and facility provision regarding the effectiveness of hand washing in COVID-19 prevention in the study area.

Findings from our study indicated that students in grade eight were 4.9 times more likely to practice proper hand washing practices compared to students in grade five. This finding was agreed with other studies conducted in Ethiopia (19, 21). The possible reason for this may be the fact that students could learn as their age is increasing and are more likely to practice what they learned. Higher grades have a positive impact on students' hand washing practice. The result from our study found that urban students were 3.5 times more likely to practice hand washing compared to students from rural residences. This was consistent with studies conducted in Ethiopia and Bangladesh (19, 20, 30) indicated that students from urban residences were more likely to practice proper hand washing. This may be due to behavioral differences among rural and urban residents and also to inadequate provision of facilities like water and soap. The presence of hand washing facilities and household level status issues is also agreed with studies conducted in other countries (19, 20). However, WHO recommends that both rural and urban settings should be targeted for hand washing practice intervention with similar emphasis.

In our study, students' having a referent (role model) as parents, teachers, or health professionals for hand washing was significantly associated with hand washing practice, whereby students whose referents were parents were 4.41 times, teachers were 3.69 times and health professionals were 3.17 more likely to practice proper hand washing compared to friends. This finding is similar to the findings of other studies conducted in Ethiopia and Indonesia (19, 20, 31). This could be due to the close monitoring of students by health professionals, parents, and teachers as a result of the COVID-19 pandemic.

The findings of our study showed that having a hand washing facility was found to be statistically significant with hand washing practice, whereby respondents who had a hand washing facility in their home were 3.62 times more likely to practice hand washing compared to their counterparts. This finding is consistent with the findings of studies conducted in Ethiopia, Vietnam and Peru (19, 32, 33), which mentioned that having a hand washing facility triggers students to wash their hands. Similarly, the results of our study found that the availability of water and soap showed a statistically significant association, with those students who had access to water and soap at home being 2.9 times more likely to practice hand washing compared with those who had no access to water and soap for hand washing. This is consistent with studies conducted in Ethiopia, Bangladesh and Columbia (20, 27, 34), which reported that the presence of water and soap showed a strong statistical association with a higher prevalence of hand washing practice. This may be due to the fact that the availability of hand-washing facilities motivates the students to wash their hands at critical moments. However, hand washing with soap was listed as one of three key behaviors in the global water supply and sanitation assessment report of 2000 by WHO that are of greatest likely benefit to health (35). The results from this study showed a low proportion of proper hand washing practice among primary students; this will increase the risk of the COVID-19 pandemic. Therefore, adequate hygiene facility provision for school children should be given emphasis to limit the COVID-19 outbreak and communicable diseases.

Findings from our study showed that being a member of a water, sanitation, and hygiene club (WASH Club) was found to be a statistically significant association with hand washing with soap. Students who were members of the school WASH club were more likely to practice proper hand washing compared to their counterparts. This is similar to a study conducted in Zimbabwe (36). This may be due to students in the WASH club receiving some training pertaining to hygiene practices in general and hand washing practices in particular as a member of the club.

### Limitation of the study

The limitation of this study was it might not indicate cause and effect relationship, due to the nature of cross-sectional study design. Moreover, there might be social desirability bias (over reporting of proper hand washing practice), since the finding was based on self-reported responses of study participants. Consistently, the results from this study may not be generalized to students in other districts or other cultural contexts.

## Conclusions and recommendations

The current study found that nearly a third of students practiced proper hand washing. The study found a statistical association between hand washing practices and students' grade level, residence, referents (role models) for hand washing, the presence of a hand washing facility, access to water and soap, and membership of the school WASH club. Furthermore, the study found that access to soap and water is needed and was seen as a constraint to proper hand washing practice. Therefore, the current study revealed that there is a need to take appropriate actions, such as intervention to increase hand-washing practice and to protect students from communicable diseases. Hand washing practices in schools should be improved by the regional government, such as the education and health bureaus, through providing hand washing facilities in schools.

## Data availability statement

The original contributions presented in the study are included in the article/supplementary files, further inquiries can be directed to the corresponding author/s.

## Ethics statement

The studies involving human participants were reviewed and approved by Haramaya University College of Health and Medical Sciences, Institutional Health Research Ethics Review Committee (Ref No. IHRERC/084/2021). Written informed consent to participate in this study was provided by the participants' legal guardian/next of kin.

## Author contributions

AB conceived the idea, collected the data, and played a major role. DM, LT, and SM contributed to data analysis, writing, and editing the document. AB, DM, LT, SM, GD, FA, AM, TG, and AG gave valuable ideas for the manuscript and revised the manuscript. All authors read and approved the final version to be published and agreed on all aspects of this work.

## Funding

This work received fund for data collection from Haramaya University College of Health and Medical Science.

## Conflict of interest

The authors declare that the research was conducted in the absence of any commercial or financial relationships that could be construed as a potential conflict of interest.

## Publisher's note

All claims expressed in this article are solely those of the authors and do not necessarily represent those of their affiliated organizations, or those of the publisher, the editors and the reviewers. Any product that may be evaluated in this article, or claim that may be made by its manufacturer, is not guaranteed or endorsed by the publisher.

## Abbreviations

AOR: adjusted odds ratio
COR: crude odds ratio
CDC: center of disease control
CSA: central statistics agency
MoE: ministry of education
MoH: ministry of health
SPSS: statistical software for social science
UNICEF: united nation children's emergency fund
WASH: water supply sanitation and hygiene
WHO: world health organization.
